# Antimicrobial Activity of Clove (*Syzygium aromaticum*) Essential Oil in Meat and Meat Products: A Systematic Review

**DOI:** 10.3390/antibiotics14050494

**Published:** 2025-05-11

**Authors:** Eduardo Valarezo, Guicela Ledesma-Monteros, Ximena Jaramillo-Fierro, Matteo Radice, Miguel Angel Meneses

**Affiliations:** 1 Departamento de Química, Universidad Técnica Particular de Loja, Loja 1101608, Ecuador; xvjaramillo@utpl.edu.ec (X.J.-F.); mameneses@utpl.edu.ec (M.A.M.); 2 Carrera de Alimentos, Universidad Técnica Particular de Loja, Loja 1101608, Ecuador; mgledesma1@utpl.edu.ec; 3 Facultad de Ciencias de la Tierra, Universidad Estatal Amazónica, Pastaza 160150, Ecuador; mradice@uea.edu.ec

**Keywords:** antibacterial activity, antimicrobial activity, beef, edible films, fish, Gram-negative bacteria, Gram-positive bacteria

## Abstract

**Background:** Clove (*Syzygium aromaticum*) essential oil is widely recognized for its potent antimicrobial properties, making it a valuable natural preservative in food products, particularly in meat and meat derivatives, where it helps extend shelf life and enhance food safety. **Methods:** This systematic review aims to evaluate the application of clove essential oil in meat and meat products, following the PRISMA 2020 methodology, to analyze its antimicrobial efficacy and its impact on the preservation of these products. The information search was carried out in the PubMed, ScienceDirect, SCOPUS, and Web of Science databases and included research articles in English published between 1999 and 2024, and 37 studies were confirmed as eligible. **Results:** Due to the heterogeneity of methodologies and concentrations evaluated, a narrative analysis was chosen, organizing the studies into three categories according to the application of the essential oil: direct addition, use in edible films and coatings, and encapsulation. The analysis included the main components of the essential oil, the activity analysis method, a concentration evaluation, storage conditions, the activities obtained, and a sensory evaluation. However, variability in methodologies and concentrations made direct comparison between studies difficult. **Conclusions:** Overall, this review confirms the effectiveness of clove essential oil in preserving meat and meat products but highlights the need to standardize its concentration and application conditions to optimize its use in the food industry.

## 1. Introduction

The meat industry, as an integral part of the global food chain, constantly faces the challenge of ensuring the quality and safety of its meat products [1]. In a context in which the demand for healthier and more sustainable foods is constantly increasing, the search for natural solutions has become a promising alternative to reducing dependence on chemical additives in the production and preservation of meat and meat products. Thus, among the natural ingredients that have attracted the attention of researchers and professionals in the meat industry are compounds of plant origin [2,3].

*Syzygium aromaticum*, commonly known as clove, is a very interesting plant species with enormous potential as a food preservative and is a rich source of antioxidant compounds [4]. Phytochemical analysis of various types of extract has revealed the presence of different chemical groups such as phenolic compounds, sesquiterpenes, and monoterpenes. Furthermore, eugenol, eugenol acetate, and caryophyllene have been reported as the main compounds present in its essential oil (EO) [5,6]. The presence of these molecules in the species *Syzygium aromaticum* strongly supports its wide range of biological activities reported in the literature [7].

Essential oils (EOs) are highly concentrated and volatile compounds extracted from various plant parts [7]. These compounds, characterized by their aromatic properties, have become the subject of interdisciplinary research ranging from organic chemistry and botany to medicine and psychology [8]. Among EOs, clove essential oil (CEO), derived from the dried buds of the clove tree (*Syzygium aromaticum*), stands out for its distinctive warm and spicy aroma. Furthermore, this oil is characterized by its richness in bioactive compounds, mainly with antimicrobial and antioxidant properties, which have been well documented [5]. The literature shows several studies on the effects of CEO on the microbiological, sensory, and nutritional quality of meat and meat products [9].

The results obtained by several authors have revealed that EOs present promising antimicrobial properties against a wide range of pathogenic and spoilage microorganisms, including bacteria, fungi, and yeasts [10]. Indeed, several studies have suggested that the active components of EO, such as eugenol, have the ability to damage the cell membranes of microorganisms, inhibiting their growth and survival [11]. These findings suggest that CEO could be an effective conservation barrier to improve the quality and microbiological safety of meat and meat products, reducing the need for synthetic chemical additives that often raise concerns about antimicrobial resistance.

Finally, this systematic review aims to compile and analyze the available scientific evidence on the application of CEO in meat and meat products, with the aim of providing a comprehensive view of its effectiveness and possible applications in a meat production context, thus producing healthier and safer foods, which is in line with the demands of current consumers [12].

## 2. Selected Studies

**Figure 1:**
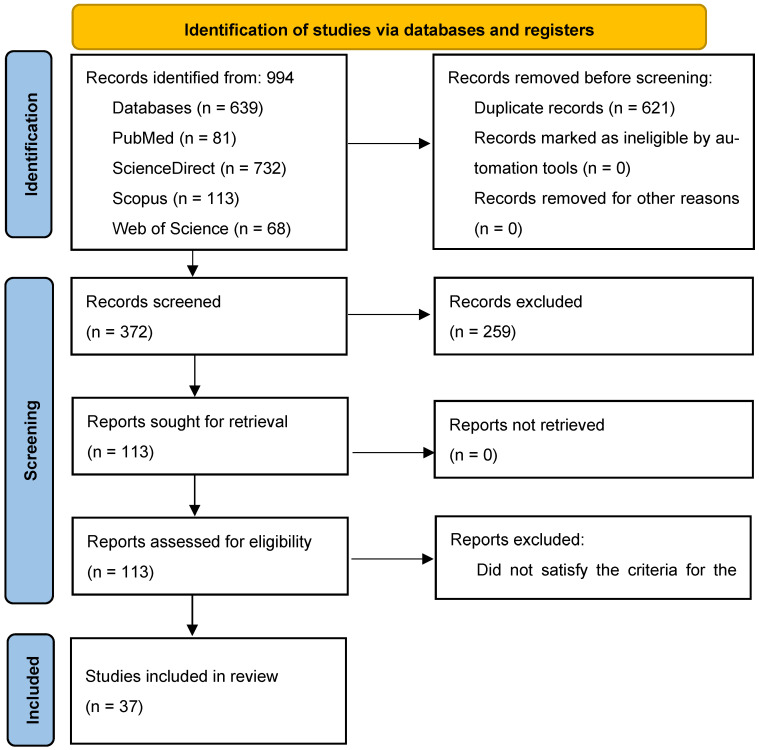
PRISMA flowchart of the study selection procedure from the reviewed article.

This systematic review was carried out following the PRISMA reporting guideline in accordance with the PRISMA 2020 statement [13]. Searches of the PubMed, ScienceDirect, Scopus, and Web of Science databases yielded 994 records. Following the removal of duplicates, 621 studies were screened by title and abstract, which yielded 113 studies for retrieval for full-text analysis. Among the excluded reports are those that only report the biological activity of the CEO (e.g., [14]); these were excluded since the EO is not tested in meat or meat products. Reports that publish the use of EOs as food supplements for slaughter animals (e.g., [15]) will also be excluded, and these reports were excluded since EO was not used directly in meat or meat products. All these reports were retrieved, and 37 were included in the final review following full-text assessments. A PRISMA flowchart [13] depicting this process is displayed in Figure 1. A total of 37 individual journal article reports were included and are available in Supplementary Information Table S1 [16,17,18,19,20,21,22,23,24,25,26,27,28,29,30,31,32,33,34,35,36,37,38,39,40,41,42,43,44,45,46,47,48,49,50,51,52].

The findings are presented according to the method of incorporation of the CEO into the food matrix, that is, through direct addition, via edible films or coatings, and as encapsulated pills. In each incorporation method section, the results of the different biological activities tested are detailed.

## 3. Incorporation Matrix of Clove Essential Oil in Meat and Meat Products

**Figure 2:**
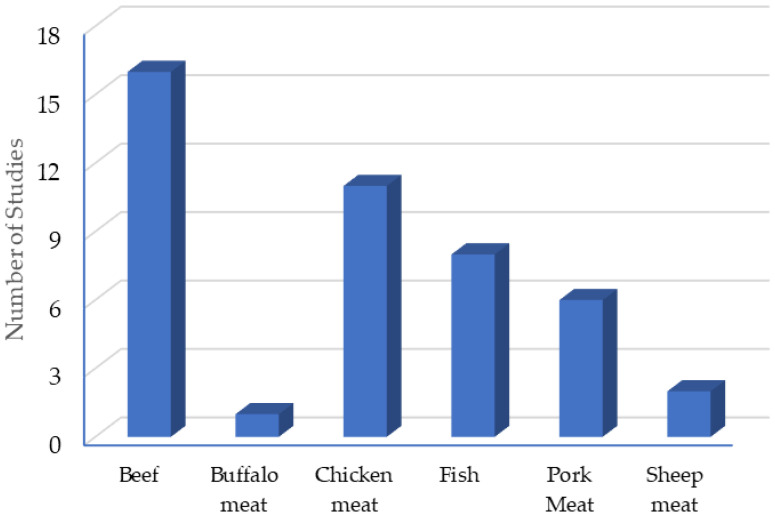
Clove essential oil application matrices.

The most common meat matrix for CEO testing is beef (16 studies). Figure 2 shows the different matrices used to test the EO of cloves and the number of studies carried out in each one. In total, 44 studies are shown, this is because in some articles, more than one matrix is used.

**Figure 3:**
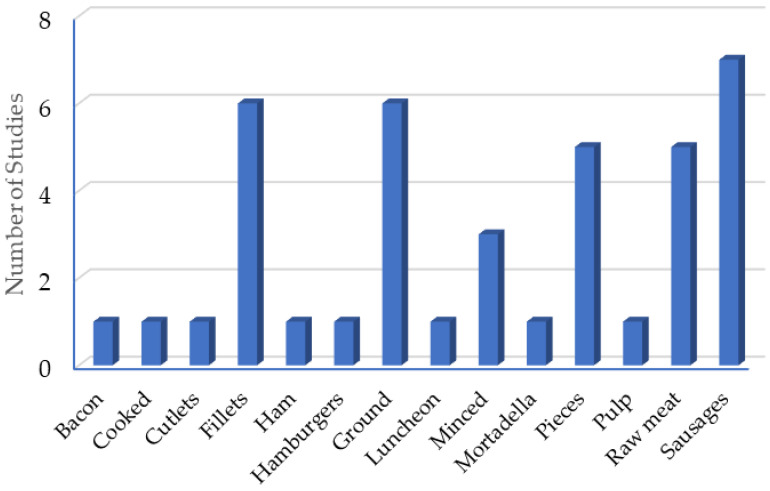
Presentation of the matrices for the application of clove essential oil.

An analysis of the presentation of the different products shows that 7 of the 37 studies were conducted on sausages (Figure 3). Of the different presentations, the hamburger, mortadella, and sausages are considered meat products, since their composition contains other ingredients besides meat.

## 4. Direct Addition of Clove Essential Oil into Meat and Meat Products

Direct addition refers to the incorporation of clove EO directly into meats and meat products. Once the EO was incorporated, antimicrobial and antifungal activity was determined. Additionally, some studies provided information on sensory evaluations.

### 4.1. Antibacterial Activity of Direct Addition

Meat is a food with high nutritional value, as it provides essential proteins, vitamins, and minerals that are required in a balanced diet. However, its preservation methods are somewhat limited when it comes to marketing it and maintaining its nutritional quality and freshness; due to its structure, it is an ideal food for the proliferation of pathogenic microorganisms. In turn, meat products have similar limitations, since meat is the main ingredient, so processing, storage, and marketing meat products must be carried out in the best way to reduce damage. The implementation of CEO arises from the proposal to implement natural preservatives due to its strong antimicrobial and antifungal activity. Table 1 shows the antibacterial activity obtained through the direct addition of CEO to meat and meat products, where the information is found in a summarized and synthesized form. The main bacteria studied were *Listeria monocytogenes*, *Escherichia coli*, *Staphylococcus aureus*, and *Bacillus cereus.*

In general, all the articles showed that the concentration of EO directly influences the inhibition of microbial growth. It was observed that in the articles where EO concentrations greater than 1% were used, the reduction in bacterial load was greater. However, as mentioned in the sensory evaluation, increasing the content had a negative impact on the taste of the samples. In turn, in most of the studies, promising results were obtained on the antimicrobial activity in vitro (commonly dilution in broth) and in vivo (meat), given that in all the samples with treatments, they had lower values in their counts in relation to the control samples. Authors such as Sharma et al. [44] attribute the reduction in these microorganisms to the changes in the environment that the oil exerted on the matrix.

According to the research by Nunes Barbosa et al. [33], Hernández-Ochoa et al. [26], Guran et al. [24], and Khaleque et al. [29], EO showed a greater inhibitory effect on Gram-positive bacteria compared to Gram-negative bacteria. The authors explain that this is due to the presence and direct interaction with the peptidoglycan layer present in Gram-positive bacteria. The membrane of Gram-negative bacteria contains lipopolysaccharides, which prevent the passage of hydrophobic molecules, creating a barrier between these microorganisms and the EO, thus obtaining favorable results of the inhibitory effect on bacteria such as *L. monocytogenes*, *S. aureus*, and *B. cereus.*

On the other hand, some authors such as Tajik et al. [48] sought to work with oils and extracts to increase their effectiveness; however, the result was not as expected. Based on their research, it was observed that the combination of CEO and grape (*Vitis vinifera*) extract generated an additive effect, so the authors recommend evaluating the synergistic effect before applying their mixture. The antimicrobial effect is greater in in vitro analyses because there are no other compounds that can intervene or influence the result, while in vivo analyses showed that there are intrinsic factors such as pH variations and macromolecules in contact, among others [44].

Clove essential oil has shown variable antimicrobial efficacy when directly applied to meat and meat products. In some studies, CEO exhibited strong antimicrobial activity against specific pathogens, whereas in others, no significant inhibition was observed. For instance, in ground beef, a 10% CEO concentration completely inactivated *Listeria monocytogenes* within three days, while a 5% concentration was ineffective throughout the storage period [29]. However, in cured ham, the addition of 10% CEO did not inhibit *L. monocytogenes* [20]. These discrepancies may be attributed to differences in the food matrix, CEO concentration, and potential protective effects of food components on microbial survival. In poultry products, CEO combined with irradiation achieved complete pathogen inactivation in minced chicken meat, whereas CEO alone reduced microbial counts in a dose-dependent manner [22]. Similarly, in chicken sausages, the CEO treatment maintained microbial counts below permissible limits; however, it was not the most effective method compared to alternative treatments [43]. Regarding beef burgers, CEO provided moderate control over aerobic and psychrophilic microorganisms, though microbial counts gradually increased over prolonged storage at −18 °C [16]. In buffalo meat burgers, CEO exhibited limited efficacy against *L. monocytogenes*, and its combination with grape seed extract did not produce a significant synergistic effect [48]. Another crucial aspect is the sensory acceptability of CEO-treated products. In several studies, high CEO concentrations, such as 10% in ground beef, were deemed unacceptable due to their strong flavor, particularly in East Asian consumer groups [29]. In beef hamburgers, CEO at 250 mg/kg was well accepted, while 500 mg/kg imparted an overpowering clove flavor, leading to lower sensory scores [16]. In pork meat and bacon, CEO combined with cinnamon oil showed no significant differences compared to commercial curing salts, indicating strong antimicrobial potential without compromising the sensory quality [46].

### 4.2. Antifungal Activity of Direct Addition

Molds and yeasts are microorganisms that can cause food degradation and spoilage, which can result in noticeable sensory changes such as bad odors, unpleasant flavors, and changes in texture. In addition, some molds and yeasts can produce toxic compounds called mycotoxins, which are dangerous to human health if ingested in significant quantities [53]. Meat is a highly perishable food due to its high water activity and favorable pH, making it an optimal medium for the proliferation of pathogenic microorganisms that cause spoilage. Habashy et al. [25] argue that the presence of mold in foods such as meat or in a meat product is due to the hygienic conditions that existed during the handling, processing, and storage process, as well as the quality of the spices that are added to the product.

Authors such as Hu et al. [54] evaluated the antifungal activity of EOs against fungi; the study showed that, although this EO has a strong and moderate activity against these microorganisms, the concentration is a key factor for their inhibition. Rana et al. [55] states that CEO presented greater antifungal activity against the genera *Aspergillus* and *Mucor* spp. and that eugenol is a powerful antifungal agent, so it is an inhibitor against all strains of fungi. However, Marchese et al. [56] establish that the effectiveness of the EO is due to the synergistic effect that occurs between eugenol and compounds of lesser proportion. Similarly, Cai et al. [57] mention that this substance causes great cellular damage in the morphology of fungi, this being its main antifungal characteristic. Table 2 shows the antifungal activity obtained via the direct addition of CEO to meat and meat products.

The antifungal activity of CEO has been evaluated in various meat products through direct application, showing variable results depending on the concentration applied and the type of product. In a study with several meat products (raw meat, fresh minced meat, ham slices, beef hamburgers, and sausages), the efficacy of CEO at concentrations between 0.5 and 1% (*v*/*w*) was evaluated against various fungal genera, including *Aspergillus*, *Penicillium*, *Cladosporium*, and others. The highest total mold count (2.85 CFU/g) was recorded in sausages, while the lowest appeared in raw meat. Regarding species distribution, *Aspergillus* predominated in all products (between 41.7% and 49%), followed by *Penicillium* (19–25.5%) and *Cladosporium* (6.3–11.9%). The 1% CEO concentration proved to be the most effective, causing significant inhibition of fungal growth. However, the treated products were considered unappetizing from a sensory perspective [25]. In the case of chicken sausages, CEO was evaluated at a concentration of 0.25% during storage at 25 °C for 15 days. The results indicated that CEO produced lower yeast and mold counts compared to control samples and other EOs evaluated. Nevertheless, from the fifth day onwards, all samples showed an unattractive appearance, unpleasant flavors, a loss of texture, and reduced juiciness, which limits its practical application from a sensory perspective [43]. The activity of CEO was also evaluated against yeasts in sheep meat at a concentration of 0.25% (*v*/*w*) during storage at 4 °C for 9 days. In this case, the inhibitory effect of CEO against yeast growth was very weak, with no significant difference compared to control samples on the third day. At the end of the testing period, samples treated with CEO obtained good scores for color and overall acceptability but low scores for odor [18]. In bonito burgers, CEO was evaluated at a concentration of 2.65 ppm during storage at 4 °C for 16 days. Initial counts of molds and yeasts were 1.3 log CFU/g, increasing to 3.40 log CFU/g in samples with CEO, indicating that it was not effective in inhibiting the growth of these microorganisms. In the sensory evaluation, no significant differences were observed in color, appearance, odor, and texture, but samples with CEO received low scores for flavor and overall acceptability [24].

The limitations in the analysis of the antimicrobial and antifungal efficacy of CEO in meat and meat products stem from the variability in the results obtained, making it difficult to generalize its effectiveness. First, the discrepancies in antimicrobial effects observed across different studies can be attributed to the influence of the food matrix, the concentration used, and the potential protective effects of food components on microbial survival. For example, while a 10% CEO concentration achieved complete inactivation of *L. monocytogenes* in ground beef [29], the same concentration showed no inhibitory effect in cured ham [20], suggesting that food composition may affect the bioavailability and efficacy of CEO. Additionally, combining CEO with other treatments, such as irradiation in poultry products, has been shown to enhance its antimicrobial action; however, this introduces variability in results and makes it difficult to attribute the effect exclusively to CEO. In terms of antifungal activity, studies show that CEO efficacy depends on the type of meat and the concentration applied, but in some cases, even at effective concentrations, a negative impact on sensory acceptability has been reported, limiting its commercial applicability.

## 5. Edible Films and Coatings Containing Clove Essential Oil

One of the main limitations in using EOs as additives is their strong aroma and prominent flavor [32]. As evidenced in the previous section, the direct application of CEO negatively influenced some attributes related to the sensory perception evaluated in the different matrices. Likewise, clove is a photosensitive and thermolabile substance, since it can easily decompose under normal environmental conditions [58]. In view of this problem, some authors investigated its EO, but through the implementation of edible films and coatings. In turn, numerous studies indicate that CEO has high antimicrobial and antifungal power (Table 1 and Table 2). Therefore, several authors proposed incorporating this additive into active packaging in such a way as to minimize the adverse effects on its direct addition, thus increasing its activity and effectiveness against deterioration reactions and microbiological growth. The main objective of this barrier technology is to minimize contact between the product matrix and its environment, thereby inhibiting bacterial growth and slowing down oxidative and other degradation reactions. Regarding the quantification of studies, a total of 37 articles that analyzed the EO through this form of application were obtained, of which 16 studies investigated antimicrobial activity and only 1 investigated the antifungal activity. Some of them studied two activities.

### 5.1. Antibacterial Activity in Edible Films and Coatings

Food is wasted every day worldwide. One of the main causes is the deterioration of its surface, which is caused by microbiological agents. The meat of an animal is highly perishable since it has a high water activity and a pH that is favorable for the development of microorganisms, so it is prone to microbiological alterations from the moment it is slaughtered. Although refrigeration temperatures are always used, from processing to the supply chain, this method has certain limitations on the shelf life of the food. It is necessary to complement it with other methods that allow the shelf life of the product to be increased, such as active films and edible coatings. Likewise, meat products are susceptible to microbiological contamination at any stage of processing and distribution. Microbial growth can cause damage to consumer health. Table 3 presents the results of research that has been carried out on the antimicrobial activity of films and edible coatings containing CEO in meat and meat products.

The results of the research showed that regardless of the food matrix and the biodegradable compounds of the films and coatings, the incorporation of CEO further improved the inhibition compared to the coated samples that did not have this bioactive compound and the control samples. In the case of beef, the research by Stoleru et al. [47] showed that the coating with polylactic acid and chitosan had a greater inhibitory effect on *Salmonella* Typhimurium, *E. coli*, and *L. monocytogenes*. On the other hand, Saricaoglu and Turhan [41] carried out their research on a fermented meat product, where they evaluated the *S. aureus* count for 45 days. The result showed minimal growth up to 30 days; however, there was no presence of coliforms in the fermented sausage. Likewise, Radha et al. [34] revealed inhibition levels of 0.65 log CFU/g and 0.57 log CFU/g for lactic acid bacteria and *Pseudomonas* spp.

Regarding pork, Jun et al. [30] obtained favorable results on the inhibition of *S. aureus* thanks to the mechanism of action of eugenol against this bacterium. According to the research by Xu et al. [59], the majority of compounds of CEO affect the cell walls of the bacteria, leading to a loss of intracellular substances and causing the death of the microorganism. In turn, Roy et al. [39] showed total inhibition against *L. monocytogenes* and slightly less in *E. coli*. Curiously, their result contradicts the findings by Stoleru et al. [47], where the antimicrobial activity was greater in *E. coli* than in *L. monocytogenes*.

Regarding chicken meat, the work by Shukla et al. [45] showed that the inhibition of a chitosan coating with CEO was greater in Gram-negative bacteria (coliforms), keeping their count below the limit (2 log CFU/g); In contrast, in Gram-positive bacteria (*S. aureus*), it crossed the limit; however, the film with the highest concentration exceeded day 35. Another article that focused on chicken meat was that by Hosseini et al. [28], where a reduction in psychrotropic bacteria, enterobacteria, and *Pseudomonas* was evident, although the film could not reduce lactic acid bacteria. However, the results presented by Requena et al. [37] revealed promising effects against *Listeria innocua* and *E. coli* in the in vitro analysis, but in the in vivo analysis, it was lower. The authors assume that this decrease is due to contact with the chemical composition of the food matrix used. In turn, Fernández-Pan et al. [21] demonstrated that mesophilic groups had lower growth than psychrophilic bacteria.

Regarding fish, Nisar et al. [32] found that an edible pectin coating with CEO at a higher concentration was more potent in reducing the growth of psychrophilic bacteria, reducing 2.3 log CFU/g. Some articles such as those by Viera et al. [51] specify that the antimicrobial activity of CEO is due to its majority compounds, mainly eugenol. Likewise, the research revealed that the oil had a strong effect against L. monocytogenes and a moderate effect against *E. coli*, *S. aureus*, and *Salmonella enterica* serovar Enteritidis. However, it was not efficient in inhibiting *Pseudomonas aeruginosa*. In contrast, Salgado et al. [40] showed greater inhibition against *Photobacterium phosphoreum* (Gram-negative) and *Brochothrix thermosphacta* (Gram-positive). Unlike the direct incorporation of oil into the matrix, films and coatings allowed for the growth of Gram-positive and Gram-negative bacteria to be inhibited. Therefore, an increase in the efficiency of the oil in terms of antimicrobial activity is shown.

Clove essential oil has proven to be an effective alternative for improving the preservation of meat and fishery products through its incorporation into edible films and coatings. In pork meat, studies indicate that CEO exhibits strong antibacterial activity against *S. aureus*, with an MIC of 1 mg/mL and an MBC of 2 mg/mL. A decrease in bacterial ATP content and an increase in APK enzyme activity were observed, suggesting damage to the microbial cell wall without affecting the sensory quality of the product [30]. In another study on pork belly, CEO at a concentration of 0.75% (*w*/*v*) completely inhibited *L. monocytogenes* and significantly reduced *E. coli* counts [39]. In beef products, CEO, whose main component is eugenol (85.7%), exhibited inhibitory effects against *L. monocytogenes*, *S.* Typhimurium, and *E. coli*, with the highest efficacy against *S.* Typhimurium [47]. In raw beef, CEO at a 3% concentration significantly reduced the growth rate of bacteria such as *Pseudomonas* spp., enterobacteria, and lactic acid bacteria [34]. In beef sucuk (fermented sausage), CEO delayed the growth of psychrotrophic bacteria and *S. aureus*, maintaining good microbiological quality for up to 45 days of storage [41]. In poultry products, the incorporation of CEO into edible coatings for chicken breast resulted in a reduction in mesophilic aerobic bacteria and *Pseudomonas* spp. during refrigerated storage for 8 days [21]. However, in another study, no antilisterial activity or total inhibition of *E. coli* was observed in PHBV-CEO coatings stored at 4 °C, although a slight reduction in bacterial counts was noted at 10 °C [37]. In chicken burgers, a 1% CEO concentration maintained coliform counts below the permissible microbiological limit for 35 days of storage, although it was ineffective against *S. aureus* [45].

In fishery products, CEO has shown variable antimicrobial activity depending on the food matrix and bacterial species. In tambaqui (*Colossoma macropomum*), CEO at concentrations of 0.08% and 0.16% exhibited moderate effects against *E. coli*, *S. enteritidis*, and *S. aureus* but was highly effective against *L. monocytogenes* [50]. In frozen tambaqui filets, CEO prevented bacterial growth for up to 120 days [51]. In rainbow trout (*Oncorhynchus mykiss*), CEO coatings inhibited the proliferation of various pathogenic bacteria, with *Shewanella putrefaciens* being the most sensitive and *Aeromonas hydrophila* being the most resistant [17]. In sardine burgers and cod filets, CEO exhibited strong activity against *P. phosphoreum* and *B. thermosphacta* but lower efficacy against *Pseudomonas* spp., *Citrobacter freundii*, and *Listeria* spp. [23,40]. From a sensory perspective, most studies report that CEO does not negatively affect the organoleptic quality of meat and fishery products at low concentrations. However, at higher concentrations, alterations in flavor and aroma have been observed. For example, in chicken burger coatings, the best acceptance was achieved with 0.5% CEO [45], while in beef filets, panelists detected sensory changes after 12 days of storage [34].

The activity of CEO in edible coatings presents inconsistencies, with some studies demonstrating significant bacterial population reductions while others report no notable inhibition, particularly against *Listeria monocytogenes* and *Escherichia coli* in certain coating materials. In fishery products, the variability in bacterial species’ response to CEO, with microorganisms such as *Shewanella putrefaciens* showing higher sensitivity while *Aeromonas hydrophila* is more resistant [17], suggests that effectiveness depends on the specific microbiota of the treated product. From a sensory perspective, although CEO at low concentrations generally does not affect organoleptic quality, at higher concentrations, its strong flavor and aroma have been rejected by consumers, particularly in products such as ground meat and hamburgers.

### 5.2. Antifungal Activity in Edible Films and Coatings

Molds and yeasts can also negatively affect the quality and visual appearance of meat products. These microorganisms can cause changes in the texture, appearance, and color of meat, resulting in a decrease in the quality perceived by consumers. In addition, the presence of molds and yeasts can contribute to product degradation over time, reducing its shelf life and affecting the consumer experience. Antifungal activity was analyzed in sardine (*Sardina pilchardus*) presented as fish patties. The microorganisms tested were *Debaryomyces hansenii*, *Aspergillus niger*, and *Penicillium expansum*. Sardine filets were obtained from a local store (Madrid, Spain). The meat was ground and mixed with salt. The mixture was divided into portions and pressed into disks. The patties were placed on acrylic plates containing the films and were stored. The concentration tested was 0.75 mL/g, and once treated, the samples were stored at 2 ± 1 °C for 13 days. The highest percentage of inhibition was in the yeast *D. hansenii* with 60.74 ± 9.90%. The authors showed that there was an antifungal effect due to the eugenol present in CEO [40]. However, unlike yeasts, molds such as *P. expansum* are more difficult to inactivate, because they are very resistant organisms despite changes in their environment [55].

## 6. Encapsulated Clove Essential Oil

Applications of CEO are limited due to its low solubility (hydrophobicity), high volatility, and instability to parameters such as light, temperature, and air (oxygen) [60]. In addition, it has limitations in terms of taste and smell, even when added directly to edible films and coatings as mentioned in the previous sections. Faced with this challenge, some authors, including Wang et al. [60], Gasti et al. [61], Radünz et al. [35], Wang et al. [52], and Rajaei et al. [36] have explored alternatives such as the encapsulation of bioactive compounds. This proposal seeks to provide greater stability for the bioactive compounds as well as a controlled release, thus allowing us to work with solutions or incorporate them directly into the meat. However, the main challenge is to find the ideal coating material to increase the biological activity of the EO.

### 6.1. Antimicrobial Activity of Encapsulated CEO

Clove essential oil has strong antimicrobial activity due to many of its compounds. However, its use is limited due to volatility and instability under environmental conditions. In addition, to completely inhibit the growth of microorganisms, it is necessary to increase its concentration, which generates alterations in the organoleptic perception of the food. For this reason, the microencapsulation of the oil is a novel alternative. The results of antibacterial activity obtained with the encapsulated CEO in meat and meat products are shown in Table 4. The analysis of the antimicrobial activity of the encapsulated CEO was analyzed using two methods: disk diffusion and minimum inhibitory concentration and minimum bactericidal concentration.

The susceptibility of the bacteria can be determined based on the size and presence of the inhibition zone. For this purpose, Arora and Kaur [62] establish that if the diameter of the zone is greater than 1.2 cm, satisfactory inhibition is achieved. According to the results of the work by Radünz et al. [35], promising results were obtained regarding the inhibition of pathogens that can be transmitted by food. The authors mention that the CEO showed an inhibitory effect on all the bacteria evaluated, and the authors assume that this antimicrobial effect is due to the lipophilic characteristics of the EO, since it causes an alteration in the permeability of the lipids of the cell membrane of the bacteria.

In turn, the EO microcapsules showed a minimum inhibitory effect on all bacteria, and the concentration of 0.304 mg/mL completely inhibited the growth of pathogens, regardless of the characteristics of their membranes. The authors assume that the action of eugenol caused a rupture of the cytoplasmic membrane in bacteria, which led to cell death, since it increased membrane permeability, causing ion extravasation (electrolyte leakage) and a loss of intracellular proteins. Likewise, the minimum bactericidal concentration was the same as the minimum inhibitory concentration. From the point of view of the mechanism of action, phenolic compounds, mainly eugenol, act by transporting protons through lipid bilayers, which causes a loss of proton motive force [36].

On the other hand, Rajaei et al. investigated the antimicrobial effect for *S. enterica* serovar Enteritidis, and they showed that there was an increase in the inhibitory effect of the free EO and the microcapsules, since the values of the minimum inhibitory concentration and the minimum bactericidal concentration of the free particles were 100 mg/mL and 200 mg/mL, respectively. In contrast, the encapsulated molecules presented lower values, specifically 5 mg/mL and 10 mg/mL. This finding indicated that there was greater interaction between the surface of the microcapsules and the cell wall of the membranes, which caused bacterial death [36].

Radünz et al. [35] made a comparison between commercial nitrite (preservative) and encapsulated CEO molecules to determine the inhibitory effect of both additives, where it was found that, regardless of the concentration, the EO was more effective in inhibiting the microbial growth of *S. aureus* than commercial nitrite. However, the authors discard the idea of completely replacing nitrite, since although this additive generates potentially carcinogenic compounds, such as nitrosamines, it also favors the formation of sensory attributes characteristic of meat products, such as aroma and color, so they recommend a combination of both additives [63].

Likewise, the results of the research by Rajaei et al. [36] revealed promising results from the first day, regardless of whether CEO was encapsulated or existed as free-form molecules: CEO reduced 3 log CFU/g of the *Salmonella enteritidis* serotype Enteritidis population. Likewise, it was found that the encapsulated particles of the EO were more effective against the free oil, since a lower concentration was needed, but it presented a greater effect throughout the test period. The authors, through in vitro and in vivo analyses, assume that the encapsulated bioactive compounds can increase their dispersion in the environment, which increases their adsorption mechanism. However, both treatments were better compared to the control samples.

### 6.2. Antifungal Activity of Encapsulated CEO

While it is true that edible films and coatings are an option to increase the shelf life of meat in general, bioactive compounds have limitations due to their volatility as well as a declining effect, since they are depleted over time, so their efficiency is not constant. For this reason, encapsulation is an alternative to protect these molecules from parameters such as light or heat, and in turn, it is possible to use them in matrices in which a thermal treatment was applied, maintaining their biological activity [52].

Mold spores are difficult to eliminate since they can withstand high temperatures or extreme pH (acids and bases) and still reproduce. Although preservatives allow us to reduce fungal growth, there are certain problems when they are subjected to high-temperature treatments, thus losing their effectiveness due to their high volatility [64]. The research by Wang et al. [52] focused on evaluating the antifungal effect of encapsulated CEO at both room temperature and cooking temperature, as well as determining the effective fungicidal concentration in both heat treatments. The result of their study revealed that as the days passed, the level of mold began to grow in the cooked samples that had low concentrations or did not contain encapsulated molecules on their surface, while the samples with higher concentrations of the EO reported a significant reduction in mold levels. Thus, it was found that the minimum effective concentration for fish was 0.07% to completely inhibit the growth of molds and spores, while for chicken and pork, it was 0.06%. Beef reported better results since its minimum effective concentration was 0.05%.

In the case of solutions with microcapsules treated at high temperatures, a decrease in the antifungal effect was evident. However, the study found that after severe thermal processing, microencapsulation provided thermal resistance to the particles, so the minimum effective concentration increased to 0.08% in each treated sample. The authors assumed that a large number of encapsulated particles maintained their antifungal activity, since the coating material, β-cyclodextrin, served as a heat-tolerant protective layer [52].

The incorporation of encapsulated CEO into meat and meat products has shown promising antimicrobial activity through different application methods, including direct addition and edible films/coatings. The antibacterial efficacy of CEO has been demonstrated in ground meat products, specifically hamburgers. When tested against common foodborne pathogens, CEO particles at concentrations of 3.04 and 0.304 mg/mL showed significant inhibition zones for *S. aureus* (2.83 cm), *E. coli* (2.81 cm), *L. monocytogenes* (2.47 cm), and *S.* Typhimurium (2.22 cm). Notably, these concentrations were effective at inhibiting *S. aureus* growth compared to commercial nitrite, demonstrating the potential of CEO as a natural preservative alternative [35]. The application of CEO through edible films and coatings has also shown effectiveness in preserving meat quality and safety. In a study with beef chops, researchers evaluated its efficacy against *S. enterica* serotype Enteritidis at concentrations of 5 and 10 μg/mL during storage at 4 °C for 12 days. Surface treatment with 100 mg free CEO, 1 mg CS-MA nanogel, and 1 and 2 mg CS-MA nanogel-encapsulated CEO resulted in significant reductions in *Salmonella* populations. The study revealed that the encapsulated CEO coating was more effective compared to its free counterpart in reducing the population of *S. enterica* serotype Enteritidis in both in vitro and in vivo assays, highlighting the benefit of encapsulation for enhanced antibacterial activity [36]. The antifungal activity of CEO was evaluated in various meat products including fish, chicken, pork, and beef. Different concentrations ranging from 0.01% to 0.1% (*w*/*w*) were tested at 37 °C for 4 days. The results revealed the presence of mold in treatments without CEO and at low concentrations. However, effective fungicidal concentrations were found to be ≤0.07% for fish, ≤0.06% for chicken and pork, and ≤0.05% for beef, demonstrating slightly different thresholds of efficacy depending on the meat type. In thermally treated coatings, the effective concentration was found to be ≤0.08% across all samples, suggesting that thermal treatment may slightly reduce the antifungal efficacy of CEO [52].

Clove essential oil encapsulation has shown promising potential by improving its stability and prolonging its antimicrobial effect; however, its effectiveness depends on the encapsulation method and interaction with the food matrix, which can influence its release and final activity. Overall, these limitations indicate that while CEO presents significant potential as a natural preservative, its application requires standardization in terms of concentration, application method, and combination with other treatments to optimize its effectiveness without compromising the sensory quality of the final product.

## 7. Research Strategy

This systematic review study aimed to evaluate the application of CEO in meat and meat products, following the guidelines of the PRISMA 2020 methodology [13]. The review was carried out following a series of methodical steps inspired by the Cochrane systematic review framework, with necessary modifications to address the specific focus of this study.

### 7.1. Information Sources

The literature search was conducted using several academic databases: ScienceDirect, SCOPUS, Web of Science (WOS), and PubMed. These databases were selected due to their comprehensive collections of scientific articles and their accessibility.

### 7.2. Search Strategy

To capture all relevant studies on the application of CEO in meat and meat products, a detailed search strategy was developed. The search terms used were “clove” OR “*Syzygium aromaticum*” AND “essential oil” AND “antimicrobial” OR “antibacterial” OR “antifungal” combined with several meat-related terms, such as “meat”, “fish”, “chicken”, and “meat products”. Boolean operators (AND, OR, and NOT), phrase search, truncation and wildcard (“*”), and field code functions were used to refine the search. The search terms may appear in the title, abstract, or keywords. The search was carried out in the databases of PubMed, SCOPUS, ScienceDirect, or Web of Science. Table 5 shows the additional information such as the literature type, language, and chronology entered according to the criteria of the researchers. This strategy allowed us to identify relevant studies.

### 7.3. Eligibility Criteria

The inclusion criteria for this review were clearly defined to ensure the relevance and quality of the selected studies. Studies that focused on the application of CEO in meat and meat products intended for human consumption were included. Research involving the use of CEO alone or in combination with other EOs was also considered. Studies that were not research articles, such as books, conference reports, editorials, etc., were excluded. Exclusion criteria included studies focusing solely on extracts other than EOs, research on animal feed supplements, and theses and dissertations unless enough articles were available. These criteria ensure that only the most relevant and high-quality studies were included in this review.

### 7.4. Study Selection

The study selection process was carried out in several stages to ensure rigor and precision. Initially, a selection was made based on the titles and abstracts of the articles identified in the search. Studies that met the inclusion criteria were selected for a full-text review. To avoid the inclusion of duplicates, reference management tools, specifically the Mendeley reference manager, were used to identify and eliminate duplicate records. Study eligibility was assessed independently by two reviewers, who reviewed the full articles. Any discrepancies between reviewers were resolved through discussion and consensus, thus ensuring the inclusion of the most relevant studies.

### 7.5. Data Extraction

Data extraction was carried out using a standardized form designed to systematically collect relevant information from each study. Eligible articles were selected by removing duplicates and excluding the articles that did not meet the inclusion and exclusion criteria. Extracted data included study characteristics such as author and year of publication. Specific information was also collected on the type of meat used and the applied concentration of the EO. This systematic approach to data extraction ensures that relevant information is collected consistently and can be adequately compared across studies.

### 7.6. Quality Assessment

Two reviewers conducted the quality assessment of all selected articles, and any differences were resolved through discussion. The quality analysis was based on the experimental design, methodology, application mechanisms, and results obtained.

### 7.7. Synthesis Methods

Initially, the number of products and presentations of the meat and meat product matrices was analyzed. Due to the nature of the results, statistical analysis was not possible; therefore, a narrative synthesis was planned. The studies were divided into three groups based on the application of the EO: direct addition, in the form of edible films and coatings, and encapsulated. Within these subgroups, the information was summarized in tables, which provide information regarding the matrix (product and presentation), main EO compounds, activity analysis method, concentration evaluated, storage conditions, results, and sensory evaluation.

### 7.8. Limitation

Our review used the PRISMA guidelines to identify as many relevant studies as possible. The search was limited to four databases recognized for their quality and contribution to research to ensure the rigor and quality of the articles included in our evaluation. The variability in methodologies and concentrations used presented a limitation, and this added to the nature of the results obtained and did not allow for the realization of a meta-analysis.

## 8. Recent Trends and Regulatory Aspects

Finally, aggregate data on the antimicrobial efficacy of CEO in meat and meat products are robust and project a potential use of the EO or its components as a natural preservative. Antimicrobial activity depends on its concentration, the food matrix, and storage conditions. Its practical application is limited by its negative effects on the sensory characteristics of products, especially flavor and odor, which affect overall consumer acceptability. Therefore, its impact on sensory properties must be carefully considered to achieve a balance between food safety and consumer acceptability. Studies with edible films and coatings, as well as studies with microencapsulated EO, are also encouraging.

Concerning regulatory aspects, the Food and Drug Administration (United States) categorizes EOs as “Generally Recognised As Safe” or “GRAS”, and CEO has been included in the group of raw EOs permitted for use in foods. Furthermore, considering the Code of Federal Regulations (CFRs), there is extensive regulation based on the definition of EOs as food additives or food contact surfaces. Finally, labeling rules have developed over time and are mandatory when it is clear that the presence of EOs has a direct impact in relation to the residual technical effects on the food placed on the market [65]. European regulations also regulate materials that come into direct contact with food (Regulation (EC) No 1935/2004) by controlling the safety and compliance of materials that come into contact with food. This regulation therefore applies to active packaging containing EOs. In parallel, Regulation (EU) No. 10/2011 was drafted with the aim of controlling nanotechnological derivatives, and it is the task of the European Food Safety Authority (EFSA) to subject foods, packaging, or additives to the relevant controls [66]. A further level of regulation is provided by two other bodies, WHO (World Health Organization) and FAO (Food and Agriculture Organization). Both institutions have created a joint committee of experts (JECFA—Expert Committee on Food Additives) that has the mandate to assess the safety of food additives, which include herbal medicines and EOs [67].

In light of these findings, the following new research trends can be proposed: (I) Further studies should focus on the action of EO blends, as well as those containing the EO of CEO, with a pleasant sensory profile that have previously tested on a consumer panel. (II) The efficacy of treatments with the volatile organic compounds (VOCs) of CEO should be considered for further research. (III) Further studies should be carried out on the effect of the main components of CEO as isolated compounds, testing again the organoleptic characteristics and palatability of processed food products.

## Figures and Tables

**Table 1:** Antibacterial activity obtained through the direct addition of clove essential oil to meat and meat products.

Matrix	Major Compounds of CEO	Microorganism	Tested Concentration	Treatment	Storage	Result	Sensory Evaluation	Ref.
Product: pork Presentation: dry-cured ham (30 g)	Eugenol (84.2%), eugenyl acetate (10.2%), and β-caryophyllene (4.2%)	*Listeria monocytogenes*	10%	EO was added to a dry-cured ham-based medium; after 1 min, *L. monocytogenes* was inoculated into the medium (10^4^ CFU/g).	The plates were incubated at 7 °C for 7 days.	CEO showed no inhibition against *L. monocytogenes*.	NR	[20]
Product: beef Presentation: mortadella (10 g)	Eugenol (80.67%)	*Clostridium perfringens*	0.066%	The EO was added to the mortadella as the ingredient and mixed with meat.	The mortadella samples were vacuum-sealed, packaged, and stored at 15 ± 1 °C for 21 days.	- The oregano, clove, and cinnamon oil mixture presented the highest antimicrobial activity, with a reduction of 1.41 log CFU/g in relation to the control. - The elimination of *C. perfringens* did not occur, but the results suggest the possibility of using EOs for the control of this microorganism.	NR	[31]
Products: beef, lamb, veal, and chicken. Presentation: Raw meat: beef, lamb, veal, and chicken; beef and chicken hamburgers; ground beef and chicken, and beef sausages.	NR	*Bacillus* spp.	0.25 and 0.5% of minced beef meat as volume/weight	Minced beef samples were mixed with Eos.	The samples were packed in polyethylene bags and stored at 4 ± 1 °C. The bacterial count was performed at 0, 3, 6, 9, 12, and 15 days.	- Bacterial count: for CEO 0.25%: day 0 = 2.939 Log10 Count (CFU/g) and day 12 = 0 Log10 Count (CFU/g); for CEO 0.5%: day 0 = 2.929 Log10 Count (CFU/g) and day 9 = 0 Log10 Count (CFU/g) - Well diffusion assay: CEO 50 mg/mL = 16 mm and CEO 100 mg/mL = 20 mm.	NR	[27]
Product: chicken Presentation: minced	Eugenol (77.32–82.36%), eugenyl acetate (8.61–10.55%), and β-caryophyllene (8.64–5.34%)	*Escherichia coli*, *Bacillus cereus*, *Staphylococcus aureus*, *Salmonella typhimurium*, and *Pseudomonas aeruginosa*	Treatment 1: 25, 50 and 100 mL/L. Treatment 2: 200, 300 and 500 mL/L. Treatment 3: EO at 3 and 5% (*v*/*w*) combined with gamma irradiation at doses of 1, 2, 3, 4, and 5 kGy.	CEO was added to samples of chicken minced meat inoculated with three pathogens.	4 ± 1 °C for 7 days	- The addition of CEO to chicken minced meat samples inoculated with three pathogens reduced the counts of these pathogens proportionally with increasing concentration. - Treatment with CEO and irradiation achieved complete inactivation of the microorganisms during the entire storage period.	NR	[22]
Product: chicken Presentation: sausages (10 g)	NR	Psychrophilic coliforms	0.25%	EO was applied on the surface of thawed meat pieces. Then, the meat was cut into small pieces and mixed with the rest of ingredients to prepare the sausages.	Storage stability testing was carried out on fresh chicken sausages for 20 days at 4 ± 1 °C.	- The microbial count remained well below the permissible limit for fresh meat products (7 log CFU/g). - However, the CEO treatment was not the most effective. With respect to the psychrophilic count, the samples with CEO showed a lower count during the entire test period. - No significant increase in coliform counts was observed.	From day 5 onwards, all samples showed an unattractive appearance, unpleasant flavors, a loss of texture, and reduced juiciness.	[43]
Product: chicken Presentation: sausages (10 g)	NR	*Escherichia coli*, *Proteus* spp., *Pseudomonas aeruginosa*, *Salmonella enterica*, *Listeria monocytogeneges*, *Staphylococcus aureus*, and *Bacillus cereus*	0.25%	EO was applied on the surface of thawed meat pieces. Then, the meat was cut into small pieces and mixed with the rest of ingredients to prepare the sausages.	Chicken sausages were vacuum packaged in LDPE–Nylon–LDPE coextruded multilayer films and stored at −18 ± 2 °C for 45 days.	- MIC (in vitro) = 0.25% for *E. coli*, *Salmonella enterica*, and *B. cereus*. - MIC (in vitro) = 0.50% for *L. monocytogenes* and *S. aureus.* - Not efficient (in vitro) for *P. aeruginosa* and *Proteus* spp. - The in vivo analysis presented moderate antimicrobial activity for total plate count and psychrophilic bacteria. On the other hand, the coliform count was observed at day 30.	Although the CEO samples had higher scores than other treatments, they did not have favorable scores with respect to overall acceptance.	[44]
Product: beef Presentation: ground (25 g)	NR	*Listeria monocytogenes*	5 and 10% of crude and commercial EOs	Crude and commercial EOs were applied by mixing with the inoculated ground beef.	Samples of ground beef contaminated with *L. monocytogenes* were packaged individually in Ziploc bags and stored in refrigeration at 8 °C, chilling at 0 °C for 7 days, as well as freezing (−18 °C) for 60 days.	- The 10% CEO could completely inactivate *L. monocytogenes* in ground beef within 3 days after inoculation. - The 5% CEO (crude and commercial) was not effective in inhibiting the bacteria during the entire storage period.	- The 5% concentration was accepted in countries from South Asia and Africa but was not liked by people from East Asia. - The 10% CEO concentration was not accepted at all due to its strong flavor.	[29]
Product: fish patties Presentation: ground	NR	Total aerobic mesophilic bacteria (TAMB), *Staphylococcus- Micrococcus*, and coliforms bacteria	2.65 ppm (the maximum daily permissible intake)	EO was added to the fish patties.	The fish patties were placed on FOA plates, wrapped with plastic wrap, and stored at 4 °C for 16 days.	- Regarding the TAMB index, although there was an increase in these bacteria, the CEO showed an inhibitory effect. - The coliform and *Staphylococcus*–*Micrococcus* spp. counts were 5.85 log CFU/g and 4 log CFU/g, respectively (microbiological analyses were only conducted up to day 10).	The analysis revealed that, in terms of color, appearance, odor, and texture, there were no significant differences between the groups. However, the samples with CEO received low scores for flavor and overall acceptability.	[24]
Product: beef Presentation: hamburgers	Eugenol (70–95%)	Aerobic microorganisms and psychrophiles	250–500 mg/kg	The patty portions were mixed with the EO, and patties were formed using a conventional patty maker.	The patties were packaged in air-permeable low-density polyethylene bags and frozen at −18 °C for 3 months.	- The APC of the samples containing CEO gradually increased during the study period. - Regarding PPC, the count varied from 3 log CFU/g to 3.73 log CFU/g in both concentrations.	For the samples with 250 mg/kg CEO, the results showed scores higher than eight in taste and odor, with no significant difference between samples (the control and with marjoram oil). In contrast, for the samples with 500 mg/kg CEO, there were strong clove flavors, so their acceptability were low.	[16]
Product: beef Presentation: pulp (10 g)	NR	*Escherichia coli*, *Bacillus cereus*, Salmonella, *Listeria monocytogenes*, *Yersinia enterocolitica*, *Campylobacter jejuni*, *Clostridium perfringens*, *Staphylococcus aureus*, and *Toxoplasma gondii*	750, 1500, and 2250 mg/L	The EO was applied to 10 g meat samples.	Meat samples were stored in a freezer at 2 °C.	- MIC = 750 uL. - The 2250 uL CEO concentration produced a reduction of 3.78 log CFU/g compared to the other concentrations.	NR	[26]
Product: buffalo Presentation: hamburgers (25 g)	Eugenol (59.97%), β-caryophyllene (15.36%), 2-methoxy-4-[2-propenyl] phenyl acetate (13.21%), and α-humulene (3.93%)	*Listeria monocytogenes*	0.1%	Ground meat was inoculated with L. monocytogenes; then, the EO was added and homogenized.	Patties were formed using a burger mold, separately packaged in sterile polyethylene bags, and stored at 8 °C for 9 days.	- The authors reported a minimal increase in the samples containing only CEO; the initial population was 5.18 log CFU/g, and the final population was 5.57 log CFU/g. - CEO alone was more effective against *L. monocytogenes*. - An additive effect was observed between CEO and grape seed extract, as it was not as efficient as expected	NR	[48]
Product: beef Presentation: ground (10 g)	Eugenol (89.80%), trans-caryophyllene (5.88%), and α-humulene (2.30%)	*Listeria monocytogenes*	1.56–3.12% and 6.25% (*w*/*v*)	Ground beef samples were incubated with *L. monocytogenes*, and the EO was added and homogenized.	The samples were stored in refrigeration at 5 ± 2 °C for 3 days.	For MIC = 1.56%, reduced colony populations were observed from the first day (at all concentrations). On the second day, no counts were detected at concentrations of 3.12% and 6.25%.	Sensory analysis showed negative scores from the minimum concentration (1.56%) due to imminent rejection at concentrations above this one.	[19]
Product: beef Presentation: hamburger	NR	*Escherichia coli*	1% (*w*/*w*)	EO was added to ground beef, mixed manually, and then inoculated with 350 µL of *E. coli* O157:H7.	Ground beef patties were prepared and cooked on a griddle set to 400 °F. The patties were immediately cooled in ice-cold water, and the analysis was performed.	CEO reduced the pathogen by 1.6 log CFU/g of the *E. coli* population	NR	[38]
Product: beef Presentation: minced (25 g)	NR	Vancomycin-resistant enterococci (VRE) and *Escherichia coli* O157:H7	0.1, 0.5, and 1% (*v*/*w*)	The minced meat samples were added at different concentrations, inoculated with microorganisms, and homogenized using a Stomacher lab blender.	The samples of minced meat were stored in high-density polyethylene bags and refrigerated at 7 °C for 14 days.	- CEO present MIC_90_ and MBC_90_ values of 2%. - The meat samples treated with clove oil showed lower counts of VRE than the corresponding control samples when maintained at 7 °C.	The minced meat that contained a higher concentration of CEO had an unpleasant taste.	[42]
Product: beef Presentation: minced (25 g)	NR	*1. Pathogenic bacterial strains (Listeria monocytogenes*, *Staphylococcus aureus*, *Escherichia coli*, *and Salmonella enteritidis).* *2. Natural microbiota of the meat: mesophilic and psychrotrophic bacteria*.	0.10% (v/g)	Minced irradiated meat samples were inoculated (10^4^–10^5^ CFU/g) with bacterial strains and EO.	Minced meat samples were packaged in individual portions and maintained at 5 °C for 7 days.	- MIC90%= 0.09% *v*/*v*. - The most effective concentration against Gram-negative strains was at 0.10% *v*/*v*, whereas against Gram-positive microorganisms, it was 0.09% *v*/*v*. - Although, in the first test, the oils were able to reduce 1.3 log CFU/g, the result was not significant. -The psychrotropic reduction tests showed no significant difference against the control sample.	NR	[33]
Product: beef Presentation: ground (25 g)	Eugenol (78%) and eugenyl acetate (13.77%)	*Salmonella* typhi	0.5 % (p/p)	Ground beef samples were inoculated with 10^6^ CFU/g of *Salmonella* Typhi. The EO was added at a concentration of 0.5% (*w*/*w*) and was homogenized by mixing.	Meat samples were packaged under air in 0.5 mil metalized polyester/2 mil ethylene vinyl acetate copolymer bags and irradiated at 4 °C with different doses from 0 to 1.75 kGy.	CEO was one of the most effective treatments for reducing *Salmonella* Typhi in ground beef due to a fourfold increase in radiosensitivity (D_10_ = 0.094 ± 0.001 kGy).	NR	[49]
Product: pork Presentation: bacon and sausage	CEO: Eugenol (76.18%) and caryophyllene (14.69%) Cinnamon EO: Cinnamaldehyde (66.86%) and caryophyllene (7.43%) EO mixtures (25:75): eugenol (55.8%) and cinnamaldehyde (20.35%) Encapsulated EO mixture (25:75): eugenol (60.1 %) and cinnamaldehyde (18.9%)	Counting of *Escherichia coli* and *Salmonella*	2 g EO/kg sausage	The ground meat was mixed with ingredients; then, the added EO was homogenized in a cutter. The EO was added in its free form and encapsulated (mixture of clove and cinnamon EO 75:25, *v*/*v*).	The fresh sausages were packed in individual low-density polyethylene packages at 4 °C for 32 days.	The free mixture of EOs showed no significant differences against commercial curing salts, indicating a strong antimicrobial potential.	NR	[46]

NR: not reported; CFU: colony-forming unit; kGy: kilograys; APC: aerobic plate count; PPC: psychrotrophic plate count; VRE: vancomycin-resistant enterococci; MIC: minimum inhibitory concentration; MBC: minimum bactericidal concentration; D_10_: radiation dose required to reduce 90% of the population.

**Table 2:** Antifungal activity obtained via the direct addition of clove essential oil to meat and meat products.

Matrix	Major Compounds of CEO	Microorganism	Tested Concentration	Storage	Result	Sensory Evaluation	Ref.
Products: beef and pork Presentation: raw meat, minced, luncheon, burgers, and sausages (125 g)	NR	*Aspergillus*, *Penicillium*, *Cladosporium*, *Alternaria*, *Fusarium*, *Mucor*, *Rhizopus*, *Sporotricum*, *Thamnidium*, *Alternania*, and *Curvularia*	0.5–1% (***v***/*w*)	4 ± 1 °C for 9 days	- The highest mean total mold (2.85 CFU/g) was recorded in sausages, while the lowest was in raw meat. - The most predominant mold species were *Aspergillus* (hamburgers 49%, ham 47.8%, fresh minced meat 46%, raw meat 42.9%, and sausage 41.7%); six species of the genera *Penicillium* (hamburgers 25. 5%, sausage 25%, ham 23.9% minced meat 20.6%, and raw meat 19%) and *Cladosporium* (raw meat 11.9%, sausage 9.7% hamburger 7.3, ham 6.5%, and minced meat 6.3%) were reported. - The best result was CEO at a concentration of 1%, as it caused significant inhibition.	They were not appetizing.	[25]
Product: chicken Presentation: sausages (10 g)	NR	Molds and yeasts	0.25%	25 °C for 15 days	The CEO had lower yeast and mold counts compared to its counter samples (control and EOs).	From day 5 onwards, all samples showed an unattractive appearance, unpleasant flavors, a loss of texture, and reduced juiciness.	[43]
Product: sheep Presentation: ground (50 g)	NR	Yeast	0.25% (*v*/*w*)	4 ± 1 °C for 9 days	The inhibitory effect of CEO against yeast growth was very weak, and there was no significant difference between the control samples on the third day.	At the end of the testing period, the samples treated with CEO had good scores for color and overall acceptability but low scores for odor.	[18]
Product: bonito Presentation: burgers	NR	Molds and yeasts	2.65 ppm	4 °C for 16 days	The initial counts of molds and yeasts were 1.3 log CFU/g, and the samples with CEO increased to 3.40 log CFU/g. Thus, it was not as effective in inhibiting the growth of these microorganisms.	The analysis revealed that, in terms of color, appearance, odor, and texture, there were no significant differences between the groups. However, the samples with CEO received low scores for flavor and overall acceptability.	[24]

NR: not reported; CFU: colony-forming unit.

**Table 3:** Antibacterial activity of clove essential oil incorporated into edible films and coatings for meat and meat products.

Matrix	Major Compounds of CEO	Microorganism	Tested Concentration	Storage	Result	Sensory Evaluation	Ref.
Product: pork Presentation: pieces (10 g)	NR	*Staphylococcus aureus*	1 and 2 mg/mL	4 °C for 7 days	- MIC = 1 mg/mL and MBC = 2 mg/mL. - These concentrations decreased the ATP content of the bacteria by approximately 48.67% and 62.31%, respectively. - The activity of the enzyme APK increased as the concentration of the oil rose, indicating that the microbial cell wall was damaged upon contact with the CEO.	The meat did not exhibit any adverse effects on its quality; rather, the CEO contributed to a glossy appearance and a good texture throughout the storage period.	[30]
Product: pork Presentation: pieces (5 g)	NR	*Listeria monocytogenes* and *Escherichia coli*	0.75% (*w*/*v*)	10 °C for 8 days	- The film with CEO exhibited significant antibacterial activity against Gram-positive bacteria. - The functional films with CEO caused complete inhibition against *Listeria monocytogenes* and a reduction of 2.19–2.77 log CFU/mL for *Escherichia coli*.	NR	[39]
Product: beef Presentation: pieces (1 cm^3^)	Eugenol (85.7%), eugenol acetate (7.9%), and *β*-caryophyllene (4.5%)	*Listeria monocytogenes*, *Salmonella* Typhimurium, and *Escherichia coli*	150 uL	7 °C for 2 days	It was observed that the coating with CEO had an inhibitory effect in the following order of susceptibility: *S. Typhimurium*, *E. coli*, and *L. monocytogenes*.	NR	[47]
Product: chicken Presentation: pieces (25 g)	Eugenol (79.4%), *β*-caryophyllene (13.36%), eugenol acetate (4.49%), and *α*-caryophyllene (1.67%)	*Pseudomonas* spp., lactic acid bacteria, psychotrophic bacteria, and enterobacteria	0.2 and 0.5% (*w*/*w*)	4 °C for 15 days	- Regarding mesophilic aerobes, the results revealed a reduction in the number of bacteria. Similarly, the coatings were effective in decreasing the number of *Pseudomonas*, psychrotrophic, and enterobacteria bacteria. In contrast, for lactic acid bacteria, none of the coatings had an effect on the inhibition of these microorganisms. - The study showed that there was no significant effect from the addition of CEO at various concentrations or under the packaging conditions.	NR	[28]
Product: chicken Presentation: burgers (75 g)	NR	*Staphylococcus aureus* and coliforms	0.25, 0.5, 0.75, and 1%	4 ± 1 °C for 35 days	- The samples wrapped with the coating containing the highest concentration of CEO (1%) had a significantly lower coliform count. The value remained below the microbiological limit for these bacteria (2 log CFU/g), even after 35 days of storage. In contrast, the count of *S. aureus* exceeded the permitted limit (2 log CFU/g) in all coatings. - However, the coating with the highest concentration crossed the limit on day 35.	Regarding the sensory analysis, the edible coating with 0.5% CEO received higher sensory scores because at this concentration, the edible coating did not inhibit the sensory attributes of the burgers. Coatings with concentrations higher than this reduced the flavor and aftertaste of the samples.	[45]
Product: tambaqui (*Colossoma macropomum*) Presentation: filets (100 g)	NR	*Escherichia coli*, *Listeria monocytogenes*, *Salmonella enterica*, *Staphylococcus aureus*, and *Pseudomonas aeruginosa*	0.08 and 0.16%	4 ± 1 °C for 3 days	- In the broth diffusion test, CEO showed a weak inhibitory effect against *P. aeruginosa* and moderate inhibition against *E. coli*, *S. enterica*, and *S. aureus*, while it showed a strong effect against *L. monocytogenes*. - In the disk diffusion test, the CEO results showed low inhibitory effects against *P. aeruginosa*, *E. coli*, *S. aureus*, and *S. enterica*. However, it showed moderate activity against *L. monocytogenes*. - The treatments were more effective against Gram-positive bacteria (*L. monocytogenes* and *S. aureus*). Films containing 0.08% CEO reduced growth after 48 h, while films with 0.16% CEO had an inhibitory effect on both bacteria after 24 h.	The attributes of color, texture, and aroma received scores of five and six (liked and liked very much). However, regarding the flavor, the scores were below four (neither like nor dislike).	[50]
Product: Wuchang bream (*Megalobrama ambycephala*) Presentation: filets (10 g)	NR	Enterobacteria, lactic acid bacteria, and *Pseudomonas* spp.	1 and 1.5% (*w*/*v*)	4 °C for 15 days.	- In the samples with CEO coatings (1% and 1.5%), the total viable count (TVC) did not exceed the acceptability limit, even after the research period, while the *Pseudomonas* counts showed gradual growth up to 5.6 and 4.6 log CFU/g, respectively. - The 1.5% CEO coatings showed slower growth in psychrophilic bacteria compared to other treatments and reduced 2.3 log CFU/g of enterobacteria. - The CEO was effective in inhibiting bacterial growth, especially in Gram-negative bacteria, while the growth of lactic acid bacteria remained stable throughout most of the storage period.	The results of the organoleptic analysis revealed that the film containing CEO did not have any negative effects on acceptability, as the filets with these coatings showed a firmer texture, less fishy odor, and stable color compared to the control samples. Although the rating of all sensory attributes decreased as the study period progressed, the final score given by the panelists was four. However, for fish samples, this can be considered acceptable.	[32]
Product: chicken Presentation: filets (10 g)	NR	*Listeria innocua* and *Escherichia coli*	13% (*w*/*w*)	4 °C and 10 °C for 6 days	- At 4 °C: No antilisterial activity nor total inhibition of *E. coli* of the PHBV-CEO films was observed. - At 10 °C: Microbial counts for *L. innocua* = approx. 6 log (CFU/g) and *E. coli* = approx. 5 log (CFU/g). The count reduction in *L. innocua* = 0.6 ± 0.1 log CFU and *E. coli* = 1.7 ± 0.2 log CFU.	NR	[37]
Product: beef Presentation: sucuk (traditional Turkish fermented sausage) (slices of 1.60 mm thick)	cymol (26.29%), α-pinene (20.65%), eugenol (17.02%), and 3-carene (11.62%)	Psychrotropics bacteria, coliforms, and *Stapylococcus aureus*	1.5% (*v*/*v*)	4 °C for 45 days	The application of CEO coatings delayed the growth of microorganisms (TVC and PCA) and improved the quality of the thermally treated sucuks during storage. The counts of *S. aureus* and psychrotrophic bacteria were significantly lower compared to the other treatments, even after 45 days of storage (TVC = 5.54 ± 0.02 log CFU/g, TPC = 4.84 ± 0.03 log CFU/g, and *S. aureus* = 2.65 ± 0.09 log CFU/g).	NR	[41]
Product: tambaqui (*Colossoma macropomum*) Presentation: filets (100 g)	NR	*Escherichia coli*, *Listeria monocytogenes*, *Salmonella enterica*, *Staphylococcus aureus*, and *Pseudomonas aeruginosa*	0.08 and 0.16%	−18 ± 1 °C for 120 days	Regardless of the CEO concentration, there was no microbial growth in any of the films with CEO during the storage period.	- The scores for the films with CEO (0.08%) were 5.4. - The scores for the films with CEO (0.16%) reached scores of 4.7. - The scores correspond to “neither like nor dislike” in both cases.	[51]
Product: rainbow trout (*Oncorhynchus mykiss*) Presentation: pieces (10 g)	NR	*Lactobacillus sakei*, *Pseudomonas fragi*, *Shewanella putrefaciens*, *Aeromonas hydrophila*, and *Vibrio alginolyticus* *Listeria innocua*, *Escherichia coli*, *Staphylococcus warneri*, *Enterococcus faecalis*, and *Leuconostoc mesenteroides*	20 g/kg	4 °C for 22 days	- The in vitro analysis of the samples coated with CEO films showed the inhibition of microbial growth in all bacteria except for *A. hydrophila*. However, there was slow antimicrobial activity against *E. faecalis*, *S. warneri*, *V. alginolyticus*, and *L. sakei*, while the most sensitive bacterium was *S. putrefaciens*, followed by *P. fragi*, *L. innocua*, *E. coli*, and *L. mesenteroides*. - The in vivo results showed that the microbial load was lower in all bacteria when combined with high pressures, including *A. hydrophila*, while cooking did not achieve the same inhibitory effect.	NR	[17]
Product: beef Presentation: filets (25 g)	eugenol (83.3%) and caryophyllene (10.6%)	*Pseudomonas* spp., enterobacteria, and lactic acid bacteria	3% (*v*/*v*)	4 °C for 15 days.	- The present study demonstrated that the use of CEO films resulted in a reduction of 0.65 log CFU/g of lactic acid bacteria in the samples. - In contrast, for *Pseudomonas* spp., the decrease was 0.57 log CFU/g. Finally, for enterobacteria, the reduction was 0.40 log CFU/g. - CEO films reduced the rate of microbial growth in raw beef.	The films containing CEO had a distinctive but pleasant taste and smell on the first day. However, the scores on the hedonic scale dropped to the rejection limits (six points) by the 12th day of the analysis.	[34]
Product: chicken Presentation: raw meat	NR	Total aerobic mesophilic bacteria, *Enterobacteriaceae*, lactic acid bacteria, and *Pseudomonas* spp.	5, 10, 20, and 30 g/kg	4 °C for 8 days	- The developed antimicrobial edible films showed high effectiveness against the main spoilage organisms developed on skinless chicken breasts stored in refrigeration for 8 days. - The results of this research have direct applications in the food industry to enhance the control of spoilage organisms such as *Pseudomonas* spp. or lactic acid bacteria.		[21]
Product: sardine (*Sardina pilchardus*) Presentation: burgers (50 g)	NR	*Lactobacillus acidophilus*, *Salmonella cholerasuis*, *Listeria innocua*, *Citrobacter freundii*, *Escherichia coli*, *Shigella sonnei*, *Pseudomonas aeruginosa*, *Yersinia enterocolitica*, *Brochothrix thermosphacta*, *Staphylococcus aureus*, *Bacillus cereus*, *Listeria monocytogenes*, *Clostridium perfringens*, *Aeromonas hydrophila*, *Photobacterium phosphoreum*, *Shewanella putrefaciens*, *Pseudomonas fluorescens*, *Vibrio parahaemolyticus*, *Bacillus coagulans*, *Bifidobacterium animalis* spp., *Bifidobacterium bifidum*, *Enterococcus faecium*, and *Lactobacillus helveticus*	0.75 mL/g	2 ± 1 °C for 13 days	- Of all the bacteria studied, the films with CEO exhibited the highest inhibition against *P. phosphoreum* and *B. thermosphacta*, which are Gram-negative and Gram-positive bacteria, respectively. - *Pseudomonas* species were the most predominant bacteria during the storage period.	NR	[40]
Product: cod (*Gadus morhua*) Presentation: filets (100 g)	NR	*Salmonella cholerasuis*, *Listeria monocytogenes*, *Shigella sonnei*, *Citrobacter freundii*, *Yersenia enterocolitica*, *Brochothrix thermosphacta*, *Bacillus cereus*, *Clostridium perfringens*, *Staphylococcus aureus*, *Pseudomonas fluorescens*, *Pseudomonas aeruginosa*, *Shewanella putrefaciens*, *Photobacterium phosphoreum*, *Listeria innocua*, *Escherichia coli*, and *Lactobacillus acidophilus*	0.75 mL/g	2 ± 1 °C for 11 days	- In terms of qualitative antimicrobial activity, CEO exhibited the greatest inhibitory effect, followed by EOs of rosemary and lavender. -The most sensitive bacteria were *C. perfringens*, *B. cereus*, and *S. aureus*, while the most resistant bacteria were *Pseudomonas* spp., *C. freundii*, *Y. enterocolitica*, and *Listeria* spp. - In terms of quantitative antimicrobial activity, CEO exhibited a percentage of inhibition of the total plate surface of 10.61 ± 0.3% for *P. phosphoreum* and 15.74 ± 1.79% for *S. putrefaciens*. - The films incorporating CEO were antimicrobial against the four microorganisms tested. The matrix G-Cl or G-Ch-Cl did not affect the antimicrobial activity. *L. acidophilus* was found to be the most sensitive to the film among the microorganisms (G-Cl = 12.76 2.51% and G-Ch-Cl = 12.60 3.42%).	NR	[23]

G-Cl: gelatinclove–clove essential oil; G-Ch-Cl: gelatine–chitosan–clove essential oil; MIC: minimum inhibitory concentration; MBC: minimum bactericidal concentration; PHBV: poly(3-hydroxybutyrate-co-3-hydroxyvalerate).

**Table 4:** Antibacterial and antifungal activity obtained from the incorporation of encapsulated clove essential oil in meat and meat products.

Activity	Matrix	Major Compounds of EO	Microorganisms	Tested Concentration	Storage	CEO Results	Ref.
	Direct Addition						
Antibacterial	Product: 57% lean beef and 10% swine fat Presentation: hamburger	Eugenol (56.06%), caryophyllene (39.63%), and α-caryophyllene (4.31%)	*Staphylococcus aureus*, *Escherichia coli*, *Listeria monocytogenes*, and *Salmonella* Typhimurium	3.04 and 0.304 mg/mL (CEO particles)	4 °C for 15 days	- The inhibition zones for *S. aureus*, *L. monocytogenes*, *S.* Typhimurium, and *E. coli* were 2.83 cm, 2.47 cm, 2.22 cm, and 2.81 cm, respectively. - The study revealed that concentrations of 3.04 mg/mL (3.84 Log CFU/g) and 0.304 mg/mL (4.47 Log CFU/g) inhibited the growth of *S. aureus* compared to commercial nitrite.	[35]
	**Edible Films and Coatings**						
	Product: beef Presentation: cutlets (10 g)	Eugenol (63.4%), caryophyllene (16%), and eugenyl acetate (13.1%)	*Salmonella enterica* Ser. Enteriditis	5 and 10 ug/mL	4 °C for 12 days	- The surface treatment of beef with 100 mg of free CEOs, 1 mg CS–MA nanogel, and 1 and 2 mg CS–MA nanogel-encapsulated CEOs resulted in significant reductions in *Salmonella* populations by 1.23, 0.58, 0.58, and 0.68 log CFU/g, respectively. - The encapsulated CEO coating was more effective compared to its free counterpart in reducing the population of *Salmonella enterica* Ser. Enteriditis in both in vitro and in vivo assays.	[36]
Antifungal	Products: beef, chicken, fish, and pork Presentation: Cooked (20 × 20 × 20 cm)	NR	Mold and mold spore levels	0.1, 0.09, 0.08, 0.07, 0.06, 0.05, 0.04, 0.03, 0.02, 0.01, and 0% (*w*/*w*)	37 °C for 4 days	- The study revealed the presence of mold in the treatments without CEO and at low concentrations. The effective fungicidal concentrations were ≤0.07% (fish), ≤0.06% (chicken and pork), and ≤0.05% (beef). - In the case of thermally treated coatings, the effective concentration was found to be ≤0.08% (in all samples).	[52]

CS-MA: chitosan–myristic acid.

**Table 5:** Keywords used for the process of finding the relevant literature.

Criterion	Eligibility	Exclusion
Literature type	Journal (research articles)	Books, book series, chapters in books, systematic review articles, and conference proceedings
Language	English	Non-English
Timeline	Between 1999 and 2024	1998 and earlier
Country/territory	World	

## Data Availability

No new data were created or analyzed in this study. Data sharing is not applicable to this article.

## Supplementary Materials

The following supporting information can be downloaded at https://www.mdpi.com/article/10.3390/antibiotics14050494/s1, Table S1: Reference list of all papers included in the review.

## Abbreviations

The following abbreviations are used in this manuscript:

APC	Aerobic plate count
CEO	Clove essential oil
CFU	Colony–forming unit
CS-MA	Chitosan–myristic acid
D10	Radiation dose required to reduce 90% of the population
EO	Essential oil
EOs	Essential oils
G-Ch-Cl	Gelatine–chitosan–clove essential oil
G-Cl	Gelatinclove–clove essential oil
Gy	Gray
MBC	Minimum bactericidal concentration
MIC	Minimum inhibitory concentration
PC	Psychrophilic count
PHBV	Poly(3-hydroxybutyrate-co-3-hydroxyvalerate)
PPC	Psychrotrophic plate count
TMAB	Total mesophilic aerobic bacteria
TPC	Total plate count
VRE	Vancomycin-resistant enterococci
