# Supplementary material for: Antimicrobial Activity of Clove (Syzygium aromaticum) Essential Oil in Meat and Meat Products: A Systematic Review
Source: Antibiotics (Basel). 2025 May 11;14(5):494. doi: 10.3390/antibiotics14050494 (PMC12108159; doi:10.3390/antibiotics14050494)
Supplement: Supplementary file 1 [file antibiotics-14-00494-s001.zip › antibiotics-3602585-supplementary.pdf]

Table S1. Reference list of all papers included in the review.

Reference	Ref. Number
Abdel-Aziz, M.E.; Morsy, N.F.S. Keeping Quality of Frozen Beef Patties by Marjoram and Clove Essential Oils. J. Food Process. Preserv. 2015, 39, 956-965. https://doi.org/10.1111/jfpp.12309.	16
Albertos, I.; Rico, D.; Diez, A.M.; González-Arnáiz, L.; García-Casas, M.J.; Jaime, I. Effect of edible chitosan/clove oil films and high-pressure processing on the microbiological shelf life of trout fillets. J. Sci. Food Agric. 2015, 95, 2858-2865. https://doi.org/10.1002/jsfa.7026.	17
Aliakbarlu, J.; Khalili Sadaghiani, S. Effect of Avishane Shirazi (Zataria Multiflora) and Clove (Syzygium Aromaticum) Essential Oils on Microbiological, Chemical and Sensory Properties of Ground Sheep Meat During Refrigerated Storage. J. Food Qual. 2015, 38, 240-247. https://doi.org/10.1111/jfq.12147.	18
De Oliveira, T.L.C.; Cardoso, M.G.; Soares, R.A.; Ramos, E.M.; Piccoli, R.H.; Tebaldi, V.M.R. Inhibitory activity of Syzygium aromaticum and Cymbopogon citratus (DC.) Stapf. essential oils against Listeria monocytogenes inoculated in bovine ground meat. Braz. J. Microbiol. 2013, 44, 357-365. https://doi.org/10.1590/S1517-83822013005000040.	19
dos Santos, L.R.; Alía, A.; Martin, I.; Gottardo, F.M.; Rodrigues, L.B.; Borges, K.A.; Furian, T.Q.; Córdoba, J.J. Antimicrobial activity of essential oils and natural plant extracts against in a dry-cured ham-based model. J. Sci. Food Agric. 2022, 102, 1729-1735. https://doi.org/10.1002/jsfa.11475.	20
Fernández-Pan, I.; Mendoza, M.; Maté, J.I. Whey protein isolate edible films with essential oils incorporated to improve the microbial quality of poultry. J. Sci. Food Agric. 2013, 93, 2986-2994. https://doi.org/10.1002/jsfa.6128.	21
Gibriel, A.Y.; Ali, H.G.M.; Abdeldaiem, M.H. Antibacterial Activity of Clove (Syzigium aromaticum L) Essential Oil and Gamma Irradiation against Some Food-Borne Pathogens in Minced Chicken Meat. Arab J. Nucl. Sci. Appl. 2017, 50, 179-193.	22
Gómez-Estaca, J.; López de Lacey, A.; López-Caballero, M.E.; Gómez-Guillén, M.C.; Montero, P. Biodegradable gelatin–chitosan films incorporated with essential oils as antimicrobial agents for fish preservation. Food Microbiol. 2010, 27, 889-896. https://doi.org/10.1016/j.fm.2010.05.012.	23
Guran, H.S.; Oksuztepe, G.; Coban, O.E.; Incili, G.K. Influence of different essential oils on refrigerated fish patties produced from bonito fish (Sarda sarda Bloch, 1793). Czech J. Food Sci. 2015, 33, 37-44. https://doi.org/10.17221/188/2014-CJFS.	24
Habashy, A.H.A.; Darwish, W.S.; Hussein, M.A.; El-Dien, W.M.S. Prevalence of different mould genera in meat and meat products with some reduction trials using essential oils. Adv. Anim. Vet. Sci 2019, 7, 79-85.	25
Hernández-Ochoa, L.; Aguirre-Prieto, Y.B.; Nevárez-Moorillón, G.V.; Gutierrez-Mendez, N.; Salas-Muñoz, E. Use of essential oils and extracts from spices in meat protection. J. Food Sci. Technol. 2014, 51, 957-963. https://doi.org/10.1007/s13197-011-0598-3.	26
Hetta, H.F.; Meshaal, A.K.; Algammal, A.M.; Yahia, R.; Makharita, R.R.; Marraiki, N.; Shah, M.A.; Hassan, H.A.M.; Batiha, G.E.S. In-vitro Antimicrobial Activity of Essential Oils and Spices Powder of some Medicinal Plants Against Bacillus Species Isolated from Raw and Processed Meat. Infect. Drug Resist. 2020, 13, 4367-4378. https://doi.org/10.2147/IDR.S277295.	27
Hosseini, M.; Jamshidi, A.; Raeisi, M.; Azizzadeh, M. Effect of sodium alginate coating containing clove (Syzygium Aromaticum) and lemon verbena (Aloysia Citriodora) essential oils and different packaging treatments on shelf life extension of refrigerated chicken breast. J. Food Process. Preserv. 2021, 45, e14946. https://doi.org/10.1111/jfpp.14946.	28

Khaleque, M.A.; Keya, C.A.; Hasan, K.N.; Hoque, M.M.; Inatsu, Y.; Bari, M.L. Use of cloves and cinnamon essential oil to inactivate Listeria monocytogenes in ground beef at freezing and refrigeration temperatures. LWT 2016, 74, 219-223. https://doi.org/10.1016/j.lwt.2016.07.042.	29
Li, J.; Li, C.; Shi, C.; Aliakbarlu, J.; Cui, H.; Lin, L. Antibacterial mechanisms of clove essential oil against Staphylococcus aureus and its application in pork. Int. J. Food Microbiol. 2022, 380, 109864. https://doi.org/10.1016/j.ijfoodmicro.2022.109864.	30
Martins, H.H.d.A.; Simões, L.A.; Isidoro, S.R.; Nascimento, S.d.S.; Alcântara, J.P.; Ramos, E.M.; Piccoli, R.H. Preservative of Essential Oil Blends: Control of Clostridium perfringens Type a in Mortadella. Braz. Arch. Biol. Technol. 2021, 64. https://doi.org/10.1590/1678-4324-2021200106.	31
Nisar, T.; Yang, X.; Alim, A.; Iqbal, M.; Wang, Z.-C.; Guo, Y. Physicochemical responses and microbiological changes of bream (Megalobrama ambycephala) to pectin based coatings enriched with clove essential oil during refrigeration. Int. J. Biol. Macromol. 2019, 124, 1156-1166. https://doi.org/10.1016/j.ijbiomac.2018.12.005.	32
Nunes Barbosa, L.; Mores Rall, V.L.; Henrique Fernandes, A.A.; Ikeda Ushimaru, P.; da Silva Probst, I.; Fernandes, A. Essential Oils Against Foodborne Pathogens and Spoilage Bacteria in Minced Meat. Foodborne Pathog. Dis. 2009, 6, 725-728. https://doi.org/10.1089/fpd.2009.0282.	33
Radha krishnan, K.; Babuskin, S.; Rakhavan, K.R.; Tharavin, R.; Azhagu Saravana Babu, P.; Sivarajan, M.; Sukumar, M. Potential application of corn starch edible films with spice essential oils for the shelf life extension of red meat. J. Appl. Microbiol. 2015, 119, 1613-1623. https://doi.org/10.1111/jam.12932.	34
Radünz, M.; da Trindade, M.L.M.; Camargo, T.M.; Radünz, A.L.; Borges, C.D.; Gandra, E.A.; Helbig, E. Antimicrobial and antioxidant activity of unencapsulated and encapsulated clove (Syzygium aromaticum, L.) essential oil. Food Chem. 2019, 276, 180-186. https://doi.org/10.1016/j.foodchem.2018.09.173.	35
Rajaei, A.; Hadian, M.; Mohsenifar, A.; Rahmani-Cherati, T.; Tabatabaei, M. A coating based on clove essential oils encapsulated by chitosan-myristic acid nanogel efficiently enhanced the shelf-life of beef cutlets. Food Packag. Shelf Life 2017, 14, 137-145. https://doi.org/10.1016/j.fpsl.2017.10.005.	36
Requena, R.; Vargas, M.; Chiralt, A. Eugenol and carvacrol migration from PHBV films and antibacterial action in different food matrices. Food Chem. 2019, 277, 38-45. https://doi.org/10.1016/j.foodchem.2018.10.093.	37
Rounds, L.; Havens, C.M.; Feinstein, Y.; Friedman, M.; Ravishankar, S. Plant Extracts, Spices, and Essential Oils Inactivate Escherichia coli O157:H7 and Reduce Formation of Potentially Carcinogenic Heterocyclic Amines in Cooked Beef Patties. J. Agric. Food. Chem. 2012, 60, 3792-3799. https://doi.org/10.1021/jf204062p.	38
Roy, S.; Priyadarshi, R.; Rhim, J.-W. Gelatin/agar-based multifunctional film integrated with copper-doped zinc oxide nanoparticles and clove essential oil Pickering emulsion for enhancing the shelf life of pork meat. Food Res. Int. 2022, 160, 111690. https://doi.org/10.1016/j.foodres.2022.111690.	39
Salgado, P.R.; López-Caballero, M.E.; Gómez-Guillén, M.C.; Mauri, A.N.; Montero, M.P. Sunflower protein films incorporated with clove essential oil have potential application for the preservation of fish patties. Food Hydrocoll. 2013, 33, 74-84. https://doi.org/10.1016/j.foodhyd.2013.02.008.	40
Saricaoglu, F.T.; Turhan, S. Performance of mechanically deboned chicken meat protein coatings containing thyme or clove essential oil for storage quality improvement of beef sucuks. Meat Sci. 2019, 158, 107912. https://doi.org/10.1016/j.meatsci.2019.107912.	41
Selim, S. Antimicrobial activity of essential oils against vancomycin-resistant enterococci (VRE) and escherichia coli O157: H7 in feta soft cheese and minced beef meat. Braz. J. Microbiol. 2011, 42, 187-196. https://doi.org/10.1590/S1517-83822010005000005.	42

Sharma, H.; Mendiratta, S.K.; Agarwal, R.K.; Kumar, S.; Soni, A. Evaluation of anti-oxidant and anti-microbial activity of various essential oils in fresh chicken sausages. J. Food Sci. Technol. 2017, 54, 279-292. https://doi.org/10.1007/s13197-016-2461-z.	43
Sharma, H.; Mendiratta, S.K.; Agrawal, R.K.; Gurunathan, K.; Kumar, S.; Singh, T.P. Use of various essential oils as bio preservatives and their effect on the quality of vacuum packaged fresh chicken sausages under frozen conditions. LWT - Food Sci. Technol. 2017, 81, 118-127. https://doi.org/10.1016/j.lwt.2017.03.048.	44
Shukla, V.; Mendiratta, S.K.; Zende, R.J.; Agrawal, R.K.; Kumar Jaiswal, R. Effects of chitosan coating enriched with Syzygium aromaticum essential oil on quality and shelf-life of chicken patties. J. Food Process. Preserv. 2020, 44, e14870. https://doi.org/10.1111/jfpp.14870.	45
Sirena, J.T.; Magro, J.D.; Junges, A.; Steffens, C.; Cansian, R.L.; Paroul, N. Characterization of free and encapsulated cinnamon and clove essential oils for enhancing fresh sausage quality: A natural substitute for synthetic preservatives. Food Bioscience 2024, 61, 104649. https://doi.org/10.1016/j.fbio.2024.104649.	46
Stoleru, E.; Vasile, C.; Irimia, A.; Brebu, M. Towards a Bioactive Food Packaging: Poly(Lactic Acid) Surface Functionalized by Chitosan Coating Embedding Clove and Argan Oils. Molecules 2021, 26, 4500.	47
Tajik, H.; Farhangfar, A.; Moradi, M.; Razavi Rohani, S.M. Effectiveness of Clove Essential Oil and Grape Seed Extract Combination on Microbial and Lipid Oxidation Characteristics of Raw Buffalo Patty During Storage at Abuse Refrigeration Temperature. J. Food Process. Preserv. 2014, 38, 31-38. https://doi.org/10.1111/j.1745-4549.2012.00736.x.	48
Turgis, M.; Han, J.; Millette, M.; Salmieri, S.; Borsa, J.; Lacroix, M. Effect of selected antimicrobial compounds on the radiosensitization of Salmonella Typhi in ground beef. Lett. Appl. Microbiol. 2009, 48, 657-662. https://doi.org/10.1111/j.1472-765X.2009.02587.x.	49
Vieira, B.B.; Carvalho, E.A.d.; Bispo, A.S.d.R.; Ferreira, M.A.; Evangelista-Barreto, N.S. Efficiency of chitosan synergism with clove essential oil in the coating of intentionally contaminated Tambaqui fillets. Semina: Ciênc. Agrár. 2020, 41, 2793-2802. https://doi.org/10.5433/1679-0359.2020v41n6p2793.	50
Vieira, B.B.; Mafra, J.F.; Bispo, A.S.d.R.; Ferreira, M.A.; Silva, F.d.L.; Rodrigues, A.V.N.; Evangelista-Barreto, N.S. Combination of chitosan coating and clove essential oil reduces lipid oxidation and microbial growth in frozen stored tambaqui (Colossoma macropomum) fillets. LWT 2019, 116, 108546. https://doi.org/10.1016/j.lwt.2019.108546.	51
Wang, Y.-F.; Jia, J.-X.; Tian, Y.-Q.; Shu, X.; Ren, X.-J.; Guan, Y.; Yan, Z.-Y. Antifungal effects of clove oil microcapsule on meat products. LWT 2018, 89, 604-609. https://doi.org/10.1016/j.lwt.2017.11.042.	52
